# Levels of oxidative stress biomarkers in seminal plasma and their relationship with seminal parameters

**DOI:** 10.1186/1472-6890-7-6

**Published:** 2007-06-01

**Authors:** Ali Khosrowbeygi, Nosratollah Zarghami

**Affiliations:** 1 Department of Biochemistry, School of Medicine, Lorestan University of Medical Sciences, Khoramabad, Iran; 2 Department of Radio Pharmacy, Drug Applied Research Center, Tabriz University of Medical Sciences, Tabriz, Iran

## Abstract

**Background:**

There is growing evidence that damage to spermatozoa by reactive oxygen species (ROS) play a key role in male infertility. The aim of the present study was to assess seminal plasma levels of total antioxidant capacity (TAC), free 8-Isoprostane and activities of catalase and superoxide dismutase (SOD) in men with asthenozoospermia, asthenoteratozoospermia and oligoasthenoteratozoospermia compared with normozoospermic males.

**Methods:**

The patients consisted of 46 men with seminal parameters abnormalities. The patients were grouped into asthenozoospermic (n = 15), asthenoteratozoospermic (n = 16) and oligoasthenoteratozoospermic (n = 15). The control group consisted of 16 healthy males with normozoospermia. Catalase activity was measured by Aebi spectrophotometeric method. Levels of TAC and SOD were measured by commercially available colorimetric assays. Level of free 8-Isoprostane was assessed by commercially available enzyme immunoassay (EIA) method. Differences between groups were assessed using Mann-Whitney U test and Kruskal-Wallis test. Coefficients of correlation were calculated using Spearman's correlation analysis. All hypothesis tests were two-tailed with statistical significance assessed at the p value < 0.05 level with 95% confidence intervals

**Results:**

Levels of catalase and TAC were significantly lower in patients than the control group. No significant changes were seen in SOD activities. Levels of free 8-Isoprostane were significantly higher in patients than the control group. Furthermore, asthenozoospermic, asthenoteratozoospermic and oligoasthenoteratozoospermic groups had significantly lower values of catalase activity and TAC when compared to normozoospermic males. Levels of free 8-Isoprostane were significantly higher in all patients subgroups than the control group. Levels of catalase and TAC were positively correlated with sperm motility and morphology. Free 8-Isoprostane levels showed an inverse correlation with sperm motility and morphology.

**Conclusion:**

Decreasing seminal plasma antioxidants levels, especially catalase and TAC, could have significant role in etiology of impaired sperm function. Measurement of 8-Isoprostane may be used as a specific biomarker for assessing oxidative stress on sperm.

## Background

In the etiology of male infertility, there is growing evidence that damage to spermatozoa by reactive oxygen species (ROS) play a key role [1,2]. Spermatozoa contain large quantities of polyunsaturated fatty acids (PUFA). Therefore, they are susceptible to ROS-induced damage. It has been suggested that ROS induce membrane lipid peroxidation in sperm [3-5]. The seminal plasma is well endowed with an array of antioxidants that act as free radical scavengers to protect spermatozoa against oxidative stress. Seminal plasma contains a number of enzymatic antioxidants such as superoxide dismutase (SOD) and catalase. In addition, it contains a variety of non-enzymatic antioxidants [6-9].

The findings on the seminal plasma catalase and SOD activities and total antioxidant capacity (TAC) are controversial. Sanocka *et al *study showed statistically significant change in activity of SOD in infertile men compared to normozoospermic samples. They also observed that the SOD activity exceeds values obtained for normozoospermic samples only in oligozoospermic males [10]. In another study Sanocka *et al *investigated activities of SOD and catalase in men with asthenozoospermia, teratozoospermia and oligozoospermia compared to normozoospermic males. Their study showed a significant elevation in intracellular activity of SOD and decreasing in catalase activity in infertile samples [11]. Zini *et al *study showed that seminal plasma activity of SOD in infertile men is significantly grater than in fertile men while catalase activity is not different between these groups [12]. The study conducted by Siciliano *et al *showed seminal plasma enzymatic (catalase and SOD) and nonenzymatic (TAC) antioxidant capacities do not alter in the asthenozoospermic specimens, whereas SOD activity is lower in oligoasthenozoospermic samples than normozoospermic males [13]. Hsieh *et al *investigation showed that there is not a significant difference in seminal plasma or sperm SOD activity between normozoospermic and oligo- or asthenozoospermic males [14]. This group also observed that activities of SOD do not correlate significantly with sperm motility and concentration. Tkaczuk-Wlach *et al *observed that whole semen SOD activity is higher in men with oligoszoospermia than those with normozoospermia [15]. Koca *et al *study showed that seminal plasma TAC in infertile asthenozoospermic and asthenoteratozoospermic males is lower than fertile men [16]. They also observed a positive correlation between seminal plasma TAC and sperm motility.

Available data on the impact of oxidative stress on sperm are based on the measurement of seminal plasma and sperm levels of malondialdehyde (MDA) by the thiobarbituric acid-reacting substance (TBARS) assay [17-25]. Recently, it has been shown that 8-Isoprostane is a specific, chemically stable, and quantitative marker of oxidative stress in vivo. 8-Isoprostane is formed in situ in cell membranes; following free radical attack on the arachidonic acid [26-28].

The aim of the present study was to assess seminal plasma levels of TAC and free 8-Isoprostane and activities of catalase and SOD in men with asthenozoospermia, asthenoteratozoospermia and oligoasthenoteratozoospermia compared to normozoospermic males.

## Methods

### Semen Samples

A case-control study was designed. Following Institutional Review Board approval, the semen samples were collected from the case and the control groups. All specimens were collected into sterile plastic containers by masturbation after an abstinence period of 3–5 days, and were analyzed within 1 h of collection. After allowing at least 30 min for liquefaction to occur, semen analysis was performed to measure sperm concentration, sperm motility and sperm morphology using Sperm Quality Analyzer IIC (SQA IIC, United Medical Systems Inc, Santa Ana, CA, USA) [29,30]. Samples with a leukocyte concentration >10^6^/ml of ejaculate and specimens with hyperviscosity were excluded from this study. The criteria for sperm normality were as follows: sperm concentration ≥ 20 × 10^6^/ml of ejaculate, sperm motility ≥ 50% and normal sperm morphology ≥ 30% [13,29,30]. The case group consisted of men with asthenozoospermia (n = 15) (age 31.33 ± 4.84 yr), asthenoteratozoospermia (n = 16) (age 34.31 ± 5.20 yr) and oligoasthenoteratozoospermia (n = 15) (age 35.75 ± 5.33 yr). The control group consisted of 16 men with normal semen parameters and proven fertility (age 32.06 ± 3.91 yr). Liquefied semen samples were centrifuged at 10000 g for 10 minutes [12,31]. The supernatant seminal plasma was then frozen at -80°C until examination.

### TAC measurement

TAC was measured by colorimetric assay [32,33]. We used commercially available colorimetric method (Randox Laboratories Ltd, UK). The frozen seminal plasma was thawed by placing the vials in a water bath at 37°C for 20 minutes and immediately assessed for its antioxidant capacity. Twenty microliters of seminal plasma was added to 1 mL of the reconstituted chromogen, 2, 2'-Azino-di-(3-ethylbenzthiazoline sulphonate) (ABTS)-metmyoglobin (10 mL vial with 10 mL of phosphate-buffered saline buffer). Twenty microliters of Trolox (6-hydroxyl-2, 5, 7, 8-tetramethylchroman-2-carboxylic acid) at a concentration of 1.71 mmol/L was used as the standard. Whereas 20 μl of deionized water was used as a blank. One milliliter of chromogen was added to the standard and blank samples. With spectrophotometer adjusted at a wavelength of 600 nm, the initial absorbance (A1) was read. Two hundred microliters of H_2_O_2 _(250 μmol/L) was then added to all tubes, and absorbance (A2) was read exactly after 3 minutes. The difference between A2 and A1 (ΔA) was calculated. The TAC of the sample was then calculated by the following formula: TAC = Concentration of the Standard × (ΔA Blank - ΔA Sample)/(ΔA Blank - ΔA Standard). The results were expressed as mM.

### SOD activity measurement

SOD activity was measured by colorimetric assay [12,31]. We used commercially available colorimetric method (Randox Laboratories Ltd, UK). This method employs xanthine and xanthine oxidase to generate superoxide radicals which reacts with 2-(4-iodophenyl)-3-(4-nitrophenol)-5-phenyltetrazoliumchloride (I.N.T) to form red formazan dye. The SOD activity is then measured by the degree of inhibition of this reaction. One unit of SOD inhibits reduction of INT by 50% under the conditions of the assay. After thawing, the seminal plasma was diluted 30-fold with 10 mM phosphate buffer, pH 7.0. Assay was performed at 37°C. Phosphate buffer was used as blank. Mixed substrate and xanthine oxidase were added into standards and sample tubes and vortexed well. With spectrophotometer adjusted at a wavelength of 505 nm, the initial absorbance (A1) was read. Final absorbance (A2) was read exactly after 3 minutes. Percentages of inhibition of standards and samples were calculated. The SOD activity was measured using calibration curve of percentage inhibition for each standard against Log_10 _of standards and SOD activity was expressed as U/ml.

### Catalase activity measurement

Catalase activity was estimated by the method of Aebi [34]. Catalase can degrade hydrogen peroxide which can be measured directly by the decrease in the absorbance at 240 nm. The hydrogen peroxide was diluted with phosphate buffer pH 7.0 and its initial absorbance was adjusted between 0.5 to 0.6 absorbance unit at 240 nm. The decrease in the absorbance was measured. One unit of catalase activity was defined as the amount of catalase which absorbed in 30 sec at 25°C. The catalase activity was then calculated from the change in absorbance and finally expressed as U/ml.

### 8-Isoprostane assessment

We assessed free form of 8-Isoprostane and only the fraction shedded to seminal plasma from cell membranes.

### Free 8-Isoprostane purification

Free 8-Isoprostane was purified by affinity chromatography method [35]. We used commercially available affinity column (Cayman Chemical, Ann Arbor, MI, USA). All samples were centrifuged at 15000 g for isolating of particulates and precipitates. Then the supernatant was diluted 1:5 with column buffer and applied to the column. Other procedures were according to the instructions provided by the manufacturer. The ethanol washed 8-Isoprostane stored at -80°C until measurement.

### Free 8-Isoprostane measurement

At first, the elution solution was evaporated to dryness using a vacuum centrifugation. Then, the concentration of free 8-Isoprostane was measured by enzyme immunoassay (EIA) method [35]. We used commercially available EIA method (Cayman Chemical, Ann Arbor, MI, USA). The procedure for the EIA was according to the instructions provided by the manufacturer. The sample volume that used was 50 μl. Absorbance was measured at a wavelength of 405 nm using enzyme-linked immunosorbent assay (ELISA) reader (STAT FAX 2100, USA). The levels of free 8-Isoprostane were presented as ng/ml. The intra-assay coefficient of variation was <10%.

### Statistical analysis

Differences between groups were assessed using Mann-Whitney U test and Kruskal-Wallis test. Coefficients of correlation were calculated using Spearman's correlation analysis. All hypothesis tests were two-tailed with statistical significance assessed at the p value < 0.05 level with 95% confidence intervals. The data were expressed as the mean ± SEM. Statistical computations were calculated using SPSS 11.5 for windows software (SPSS Inc, Chicago, IL, USA).

## Results

Seminal parameters of the subjects are reported in Table 1. Table 2 shows comparison of seminal plasma levels of free 8-Isoprostane and TAC and activities of catalase and SOD between patients and control groups. Levels of catalase and TAC were significantly lower in patients than the control group. No significant changes were seen in SOD activities. Seminal plasma levels of free 8-Isoprostane were significantly higher in patients than the control group. Table 3 shows seminal plasma levels of free 8-Isoprostane and TAC and activities of catalase and SOD in subgroups of the patients compared to normozoospermic subjects. Asthenozoospermic, asthenoteratozoospermic and oligoasthenoteratozoospermic males had significant lower values of catalase and TAC compared with normozoospermic males. Levels of free 8-Isoprostane were significantly higher in all patients than the control group.

**Table 1:** Seminal parameters in controls and subgroups of patients.

Diagnosis	Concentration (10^6^/ml)	Motility (%)	Morphology (%)
Controls			
Normozoospermic (n = 16)	97.06 ± 5.38	57.81 ± 1.39	39.62 ± 1.04
Patients			
Asthenozoospermic (n = 15)	62.40 ± 1.90	46.0 7 ± 0.30	31.07 ± 0.25
Asthenoteratozoospermic (n = 16)	39.00 ± 2.75	36.75 ± 1.38	23.56 ± 0.89
Oligoasthenoteratozoospermic (n = 15)	13.73 ± 1.19	18.53 ± 1.28	16.80 ± 0.33

Data are reported as mean ± SEM.

**Table 2:** Seminal plasma levels of free 8-Isoprostane and total antioxidant capacity (TAC) and activities of catalase and superoxide dismutase (SOD) in controls and total patients.

Variables	Controls (N = 16)	Patients (N = 46)
TAC (mM)	1.63 ± 0.08	1.05 ± 0.04^a^
Catalase (U/ml)	22.58 ± 2.20	14.40 ± 0.93^b^
SOD (U/ml)	5.89 ± 0.96	5.32 ± 0.56
8-Isoprostane (ng/ml)	2.60 ± 0.38	18.23 ± 3.56^c^

Data are reported as mean ± SEM.

^a^p = 0.001, ^b^p = 0.0.03, ^c^p = 0.0001 in comparison with controls.

**Table 3:** Seminal plasma levels of free 8-Isoprostane and total antioxidant capacity (TAC) and activities of catalase and superoxide dismutase (SOD) in controls and subgroups of patients.

Diagnosis	8-Isoprostane (ng/ml)	TAC (mM)	Catalase (U/ml)	SOD (U/ml)
Controls				
Normozoospermic (n = 16)	6.95 ± 2.10	1.63 ± 0.08	22.58 ± 2.20	5.89 ± 0.96
Patients				
Asthenozoospermic (n = 15)	14.66 ± 4.10^a^	1.12 ± 0.06^d^	13.76 ± 1.64^e^	4.82 ± 0.54
Asthenoteratozoospermic (n = 16)	16.71 ± 5.58^b^	1.02 ± 0.08^d^	16.66 ± 1.46^f^	6.24 ± 1.50
Oligoasthenoteratozoospermic (n = 15)	23.42 ± 8.36^c^	1.01 ± 0.09^d^	12.61 ± 1.65^g^	4.82 ± 0.39

Data are reported as mean ± SEM.

^a^p = 0.01, ^b^p = 0.008, ^c^p = 0.04, ^d^p = 0.0001, ^e^p = 0.004, ^f^p = 0.04, ^g^p = 0.001 in comparison with normozoospermic men.

Then, we examined the correlation between seminal parameters and seminal plasma levels of 8-Isoprostane, TAC, SOD and catalase in total case group. Levels of TAC showed a positive correlation with sperm motility (r = 0.45, p < 0.05) and morphology (r = 0.45, p < 0.05). We also observed a direct correlation between catalase activity and sperm concentration (r = 0.41, p < 0.05), sperm motility (r = 0.41, p < 0.05), and sperm morphology (r = 0.42, p < 0.05). Seminal plasma levels of free 8-Isoprostane showed an inverse correlation with sperm motility (r = -0.26, p < 0.05) and sperm morphology (r = -0.27, p < 0.05). Levels of SOD did not show any correlation with seminal parameters.

## Discussion

The most relevant findings of this study were (i) significant elevation of seminal plasma levels of free 8-Isoprostane and decreasing of catalase and TAC levels in asthenozoospermic, asthenoteratozoospermic, oligoasthenoteratozoospermic samples compared with normozoospermic men (ii) both catalase and TAC showed a positive correlation with sperm motility and morphology while an inverse correlation was seen with free 8-Isoprostane levels.

Our findings about activities of SOD were contradicted the results of Sanocka *et al *[10,11]. Our findings confirm Sanocka *et al *results about catalase activity in infertile men [11]. Siciliano *et al*. evaluated antioxidant capacity of seminal plasma in asthenozoospermic and oligoasthenozoospermic specimens with normal viscosity and hyperviscosity [13]. Their study showed that in semen with normal viscosity levels of catalase, SOD and TAC do not alter in asthenozoospermic specimens compared with normozoospermic men. They observed that SOD activity declines in oligoasthenozoospermic males. In contrast to Siciliano *et al*., we observed a significant decrease in catalase activity and TAC in men with asthenozoospermia compared to normozoospermic men. Our finding about SOD activity in asthenozoospermic men was similar to Siciliano *et al *study. In another study, Hsieh *et al *evaluated SOD activities in seminal plasma and spermatozoa in infertile men with normozoospermia and oligoasthenozoospermia [14]. They observed that SOD activities of seminal plasma and sperm in both groups are nonsignificantly different. Hsieh *et al *also observed that SOD activities of seminal plasma and sperm are positively but nonsignificantly correlated with sperm motility and concentration. Our results about the SOD activity of the seminal plasma in oligoasthenozoospermic men were similar to Hsieh *et al *study. Koca *et al *evaluated TAC in infertile asthenozoospermic and asthenoteratozoospermic men compared to normozoosperic fertile men [16]. Their study showed that asthenozoospermic and asthenoteratozoospermic males have significantly lower mean TAC value than the control group. Koca *et al *study also showed that TAC correlates positively to sperm motility. We also observed these findings. Tkaczuk-Wlach *et al *evaluated activity of SOD in the whole semen of patients with oligozoospermia compared to patients with normozoospermia [15]. Their study showed that oligozoospermia males have significantly higher mean SOD activity than the control group. However, the finding of Tkaczuk-Wlach *et al *study was limited by the fact that they used whole semen sample, because the membrane-bound oxidases or antioxidants associated with cellular debris and/or organelles can influence activities of antioxidant enzymes such as SOD and catalase [12].

Immature spermatozoa with abnormal morphology and cytoplasmic retention are the most sources of ROS production in semen. This has been confirmed by Gil-Guzman *et al *study [36]. Their study showed that there is a direct significant correlation between ROS levels and the rate of abnormal forms in semen. Gil-Guzman *et al *also observed that there is an inverse significant correlation between seminal plasma TAC and ROS levels. They suggested that the inverse correlation between TAC and ROS might be associated with an increase in the consumption of soluble, non-enzymatic antioxidants in seminal plasma which is resulted from over production of ROS. In our study the correlation between TAC and sperm morphology was positive. According to Gil-Guzman *et al *study, this finding could be interpreted that in semen with high rate of abnormal forms, because of high levels of ROS production, consumption of non-enzymatic antioxidants will be higher.

The inverse correlation between lipid peroxidation and sperm motility has been shown by Keskes-Ammar *et al *[24]. In our study both TAC and catalase, two defenses against ROS, showed direct correlation with sperm motility. Keskes-Ammar *et al *and our study might suggest that the higher level of antioxidant status prevents lipid peroxidation in spermatozoa and therefore results in higher sperm motility. Hsieh *et al *observed a slightly positive correlation between seminal plasma SOD activity and sperm concentration [14]. Their interpretation was that higher concentrations of spermatozoa might produce higher levels of SOD. The positive significant correlation between seminal plasma catalase activity and sperm concentration that observed in our study may be interpreted similar to Hsieh *et al*. Immature spermatozoa generate primary superoxide anion. This anion is dismuted to hydrogen peroxide by SOD activity. Detoxification of hydrogen peroxide is carried out by catalase activity. Hydrogen peroxide is the primary toxic ROS for human spermatozoa that its high concentration induces lipid peroxidation and results in cell death. Therefore, the balance of the SOD and catalase activities in semen is important for maintaining sperm motility [14].

Our results are agreed with some previous studies that show increasing of lipid peroxidation by measuring MDA in sperm and seminal plasma in males with asthenozoospermia, asthenoteratozoospermia and oligoasthenoteratozoospemia [20,23]. Similar to MDA [18,24] 8-Isoprostane also showed an inverese correlation with sperm motility.

MDA is widely used index of lipid peroxidation due to its simplicity. The TBARS test application to body fluids and tissue samples is unreliable. Application of a gas chromatography/mass spectrometry (GC/MS) assay for MDA has indicated that the commonly used TBARS assay overestimates the actual MDA levels by more than 10-fold, possibly resulting from cross reactivity with other aldehydes and the harsh conditions used in sample preparation [26].

Recent studies have focused on 8-Isoprostane, as an index of lipid peroxidation. Isoprostanes are formed in situ in cell membranes; following free radical attack on the arachidonic acid. Unlike prostaglandins, which are formed from arachidonic acid following its release from the *sn*-2 position of phospholipids by phospholipase A_2_, isoprostanes are formed initially in situ, where they may contribute to the effects of oxidative stress on membrane biophysics. Measurement of 8-Isoprostane may provide a reliable marker of lipid peroxidation in vivo, because, it is a stable compound. In addition, 8-Isoprostane is specific product of free radical-induced lipid peroxidation. 8-Isoprostane has also been found to be present in detectable quantities in all normal biological tissues and in free form in all normal biological fluids. This is important because it allows the definition of a normal range such that small increases in its formation can be detected in situations of mild oxidant stress. Finally, the levels of 8-Isoprostane is unaffected by lipid content of the diet [26,28].

Evidence is beginning to emerge suggesting that isoprostanes are not only markers of oxidative injury, but active participants in the pathophysiology of some disorders. The capacity of isoprostanes to readily esterify to cell lipid membranes, and the resulting marked distortion of membrane structure and function, undoubtedly contribute to their pathophysiologic potential. As well, the existence of specific receptor for isoprostanes has been proven [37]. So, because isoprostanes are biologically active, they may have significant role in the etiology of some sperm function abnormality.

## Conclusion

It is concluded that decreasing seminal plasma antioxidant status, especially catalase activity and TAC, may have significant role in the etiology of impaired sperm function. Measurement of 8-Isoprostane may be used as a specific biomarker for assessing oxidative stress on sperm. However, further studies with a larger sample size are required to confirm these findings.

## Competing interests

The author(s) declare that they have no competing interests.

## Authors' contributions

Ali Khosrowbeygi carried out all of the experiment and participated in data analyzing and writing the manuscript.

Nosratollah Zarghami designed the study and participated in data analyzing and writing the manuscript.

## Pre-publication history

The pre-publication history for this paper can be accessed here:


